# Supplementary material for: Resolving the full spectrum of human genome variation using Linked-Reads
Source: Genome Res. 2019 Apr;29(4):635–45. doi: 10.1101/gr.234443.118 (PMC6442396; doi:10.1101/gr.234443.118)
Supplement: Supplemental Material [file supp_gr.234443.118_Supplemental_longranger-2.2.zip › longranger-2.2.tar.bz2!/lr2.2-public/lib/stan/cmdstan/stan/doc/api/html/rev_2numeric__limits_8hpp.html]

Stan: src/stan/agrad/rev/numeric\_limits.hpp File Reference


|  |  |
| --- | --- |
|  | Stan  2.5.0  probability, sampling & optimization |


- Main Page
- Related Pages
- Namespaces
- Classes
- Files

- File List
- File Members

All Classes Namespaces Files Functions Variables Typedefs Enumerations Enumerator Friends Macros Pages

- src
- stan
- agrad
- rev

Classes |
Namespaces |
Functions

numeric\_limits.hpp File Reference

`#include <limits>`  
`#include <stan/agrad/rev/var.hpp>`

Go to the source code of this file.

|  |  |
| --- | --- |
| Classes | |
| struct | std::numeric\_limits< stan::agrad::var > |
|  | Specialization of numeric limits for var objects. More... |
|  | |

|  |  |
| --- | --- |
| Namespaces | |
|  | std |
|  | Template specification of functions in std for Stan. |
|  | |

|  |  |
| --- | --- |
| Functions | |
| int | std::isnan (const stan::agrad::var &a) |
|  | Checks if the given number is NaN. More... |
|  | |
| int | std::isinf (const stan::agrad::var &a) |
|  | Checks if the given number is infinite. More... |
|  | |


---

[ Stan Home Page ]

*© 2011–2014,
Stan Development Team.*
